# RAGE: A novel target for breast cancer growth and metastasis

**DOI:** 10.18632/oncoscience.294

**Published:** 2016-03-03

**Authors:** Mohd W. Nasser, Dinesh K. Ahirwar, Ramesh K. Ganju

**Affiliations:** Department of Pathology, The Ohio State University, Columbus, OH, USA

**Keywords:** RAGE, S100A7, TAMs, TNBC

Metastasis is a major cause of mortality in Breast Cancer (BC) patients in part due to lack of clinically established targeted therapies. Among the different types of BC, triple negative BC (TNBC) (ER-, PR-, HER2-) has been associated the most with poor prognosis and survival due to early metastasis to other organs and a lack of clinically established targeted therapies. Hence, elucidating novel mechanisms that regulate metastasis would lead to the development of targeted therapies and new treatments for TNBC and metastatic breast cancers.

It is now well accepted that solid tumors, including those in the breast, have an inflammatory microenvironment. Receptor for advanced glycation end products (RAGE) is a member of the immunoglobulin superfamily of cell surface molecules which has been associated with chronic inflammation, which in turn enhances the progression of various cancers [1]. We have shown that RAGE is expressed in a panel of aggressive BC cell lines and TNBC tissues [3]. High RAGE expression was also observed in lymph node and distant metastases patient samples. In addition, we observed that high RAGE expression was associated with poor prognosis in breast cancer [3]. RAGE has also been shown to play important role in various cancers including pancreatic cancer. Under hypoxic environment, RAGE was shown to interact directly to mutant KRAS and upregulate HIF1α that leads to the development of pancreatic cancer [2].

We have shown that RAGE deficiency inhibits the growth of murine breast cancer tumor cells [3]. RAGEdeficient mice have shown less chemically-induced inflammation [1]. Furthermore, we have shown that RAGE neutralizing antibody treatment significantly inhibited breast cancer metastasis in an intracardial mouse model [3]. RAGE is a multi-ligand receptor and binds to several inflammatory ligands such as advanced glycation end products (AGE), high mobility group box 1 peptide (HMGB-1), amyloid-β peptide and the S100 family of proteins. Our mechanistic investigation has revealed that RAGE mediates its functional effects in breast cancer by binding to S100A7 [3]. S100A7 is a small molecular weight calcium-binding protein [4]. Although a number of putative functions have been proposed for S100A7, its biological role, particularly in BC, remains to be defined [4]. Phylogenetic analyses have shown the mouse ancestor mS100a7a15 to be most related to human S100A7 [5]. It has been shown that mS100a7a15 is up-regulated during carcinogen-induced mammary tumorigenesis. However, the direct functional role of mS100a7a15 in disease progression is not well-characterized. We have shown that mS100a7a15 overexpression induced hyperplasia in mammary glands of these transgenic mice [5]. Upon binding to ligands, RAGE activates its downstream signaling mechanisms that augment and maintain chronic inflammatory conditions [1]. We have also shown that S100A7 enhances NF-kB activation and its nuclear translocation in TNBC cells. These features of RAGE make it an ideal candidate for therapeutic strategies against inflammation induced cancers.

The link between inflammation and cancer has been postulated for a long time because of the presence of inflammatory cells in the tumor microenvironment, including myeloid cells and tumor associated macrophages (TAMs) [6]. These cells have been shown to significantly enhance tumor growth and the metastasis of various tumors and are key inhibitors of anti-tumor immunity [6]. In addition, collaborative interactions of tumor cells with TAMs have been associated with poor prognosis in BC [6]. TAMs have also been shown to play an important role in enhancing metastasis and drug resistance [6]. Therefore, inhibition of TAM accumulation and recruitment is a promising strategy for enhancing the effectiveness of immune-based therapies against tumors [6]. We and others have shown that RAGE is also expressed on immune cells [3, 5, 7]. RAGE expression was detected in different cell types present within the tumor microenvironment including macrophages. In addition, RAGE has been shown to be present in the microglia in the gliomas. Chen et al (2014) have shown that RAGE signaling in glioma TAMs regulates angiogenesis most likely by MMP9 [8]. We also showed in breast cancer mouse model that blocking of RAGE signaling significantly inhibited tumor growth, metastasis and angiogenesis through inhibition of MMP9+ TAMs [3]. In addition, RAGE also binds to S100A8/A9 to recruit myeloid derived suppressor cells (MDSCs) and thereby enhance cancer growth and metastasis [7]. These studies suggest that RAGE could enhance tumor growth and metastasis through regulating tumor microenvironment.

**Figure 1:**
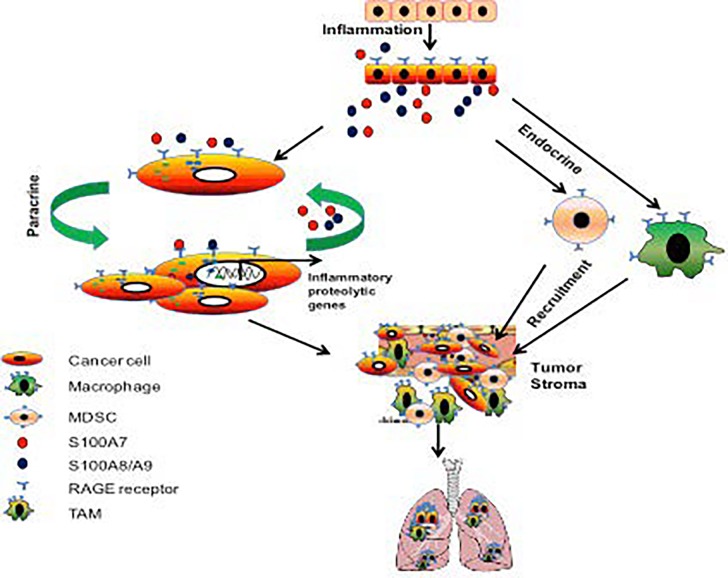
Role of S100A7/RAGE-signaling axis in breast cancer growth and metastasis

Overall, our results indicate that RAGE could be used as a novel therapeutic target and biomarker for TNBCs. Furthermore RAGE/S100A7 signaling through paracrine and endocrine mechanisms in epithelial as well as TAMs plays an important role in linking inflammation to breast cancer development and metastasis (Fig. 1). In addition, RAGE also binds to S100A8/A9 to recruit myeloid derived suppressor cells (MDSCs) and thereby enhance breast cancer growth and metastasis.
